# Development of an action plan for acute food protein–induced enterocolitis syndrome in Japan

**DOI:** 10.1016/j.waojou.2023.100772

**Published:** 2023-05-09

**Authors:** Yuri E. Kram, Miori Sato, Kiwako Yamamoto-Hanada, Kenji Toyokuni, Satoko Uematsu, Takahiro Kudo, Yoshiyuki Yamada, Yoshikazu Ohtsuka, Kenji Matsumoto, Katsuhiro Arai, Tatsuki Fukuie, Ichiro Nomura, Yukihiro Ohya

**Affiliations:** a Allergy Center, National Center for Child Health and Development, Tokyo, Japan.; b Division of Emergency and Transport Services, National Center for Child Health and Development, Tokyo, Japan.; c Department of Pediatrics, Juntendo University Faculty of Medicine, Tokyo, Japan.; d Department of Pediatrics, Tokai University School of Medicine, Kanagawa, Japan.; e Department of Allergy and Clinical Immunology, National Research Institute for Child Health and Development, Tokyo, Japan; f Division of Gastroenterology, National Center for Child Health and Development, Tokyo, Japan.

**Keywords:** Food protein–induced enterocolitis syndrome, Action plan, Delphi method

## Abstract

Reports of food protein–induced enterocolitis syndrome (FPIES) in Japan have been increasing. However, the disease itself and the treatment options are poorly understood by both patients and medical professionals. The objective of this study is to develop an action plan for acute FPIES in Japan. We prepared a single–sheet action plan that describes the management of acute FPIES episodes for caregivers on one side and medical professionals on the reverse side. To evaluate the content of the action plan, we distributed a questionnaire to caregivers of patients with FPIES and to physicians who would encounter patients with FPIES. Changes to the FPIES action plan were made based on the feedback from the participants. The Delphi method was utilized to finalize the action plan. The participants of the initial survey found the action plan to be useful but the process for determining severity to be impractical. After discussion, the authors made appropriate improvements. By the Delphi method, consensus was reached on the revised FPIES action plan. In conclusion, this Japanese FPIES action plan was created by physicians from multiple subspecialties and caregivers of patients with FPIES. The action plan may improve the management of acute FPIES reactions in the Japanese community.

## Introduction

Food protein–induced enterocolitis syndrome (FPIES) is a non–IgE–mediated gastrointestinal food allergy. The innate immune activation has been proposed as an etiology, but the detailed pathophysiology is still unknown.^1^ Acute FPIES symptoms include repetitive vomiting and diarrhea following trigger food ingestion. Severe cases warrant emergent management as profound vomiting results in dehydration and hypovolemic shock which could be life-threatening, especially in very young children.^2^ The number of reports of non–IgE–mediated gastrointestinal food allergy has increased in the last decade in Japan,^3^ and the prevalence of the disease in infants was recently reported to be 0.5% in Japan.^4^

Although the prevalence of FPIES in Japan seems to be increasing, the disease itself and the treatment options are poorly understood by both patients and medical professionals in Japan, due to the relatively recent history of the FPIES diagnosis. In Japan, an action plan for IgE–mediated food allergy is widely used,^5^ but there is no action plan for FPIES. Therefore, to aid caregivers of patients with acute FPIES reaction to know what to do based on symptom severity and to receive appropriate treatment at medical facilities, we developed an action plan for acute FPIES in Japan.

## Methods

This study was approved by the Institutional Review Board of the National Center for Child Health and Development (NCCHD). We obtained informed consent from the study participants.

### Development phase

Based on the international consensus guidelines of FPIES,^2^ we prepared a double-sided, single–sheet action plan in Japanese that describes the management of acute FPIES episodes based on severity for caregivers on one side, and for medical professionals on the reverse side. We excluded treatment with ondansetron from the action plan since this medication is not approved for FPIES in Japan. For the medical professionals’ side, we also included brief background information about FPIES with a link and a quick response (QR) code to a website that describes FPIES in detail by the Japan Intractable Diseases Information Center. The bottom of the action plan included a statement to treat IgE-mediated allergic reaction accordingly in case patients present with both acute FPIES symptoms and IgE-mediated allergic symptoms at the same time. We first discussed the content of the action plan among the physicians in the Allergy Center, NCCHD. Then we gained input from physicians in the division of pediatric emergency medicine and general pediatrics at the same institution and nurses working at the outpatient clinic of the Allergy Center. Finally, the physicians in the Eosinophilic Gastrointestinal Disorders Research Group, Policy Planning and Evaluation for Rare and Intractable Diseases, Ministry of Health, Labor and Welfare reviewed the action plan, and corrections were made. The preliminary FPIES action plan was created (Supplemental Fig. 1).

To evaluate this action plan, we recruited caregivers of patients who were diagnosed with or clinically suspected to have FPIES and a convenience sample of physicians from multiple hospitals in several regions of Japan who were likely to encounter pediatric patients with FPIES from June 2021 to October 2021. The caregivers were recruited either at outpatient clinic or during hospitalization for oral food challenge at the NCCHD, or via the online FPIES class conducted by the NCCHD for caregivers of patients who were seen at other institutions across Japan. We distributed A4-sized paper copies of the FPIES action plan with a link to an online anonymous survey to caregivers and some physicians. For the caregivers who attended the online FPIES class and the rest of physicians, an electronic file of the action plan with the link to an online anonymous survey were emailed.

Caregivers were asked to evaluate the “For Patients” side of the action plan, and physicians were asked to evaluate the “For Medical Professionals” side. Evaluations incorporated the Consumer Information Rating Form (CIRF), which was developed and validated to assess the comprehensibility, utility, and design quality of written medical patient information by consumers,^6^^,^^7^ and a modified version of the CIRF was used to develop other action plans.^8^^,^^9^ An adapted CIRF was translated into Japanese for this study. Other factors that were assessed included usefulness of the action plan, improvements in participants’ knowledge, and understanding of FPIES, and likelihood of using the action plan in the future. To assess the health literacy of the caregivers, we used the Communicative and Critical Health Literacy scale which was developed to subjectively evaluate the ability to collect, understand, and utilize the information. The score ranges from 1 to 5, with a higher score indicating higher health literacy.^10^ Demographic information of participants and free comments regarding the action plan were also collected. Caregivers and physicians could make comments on both sides of the action plan.

**Fig. 1:**
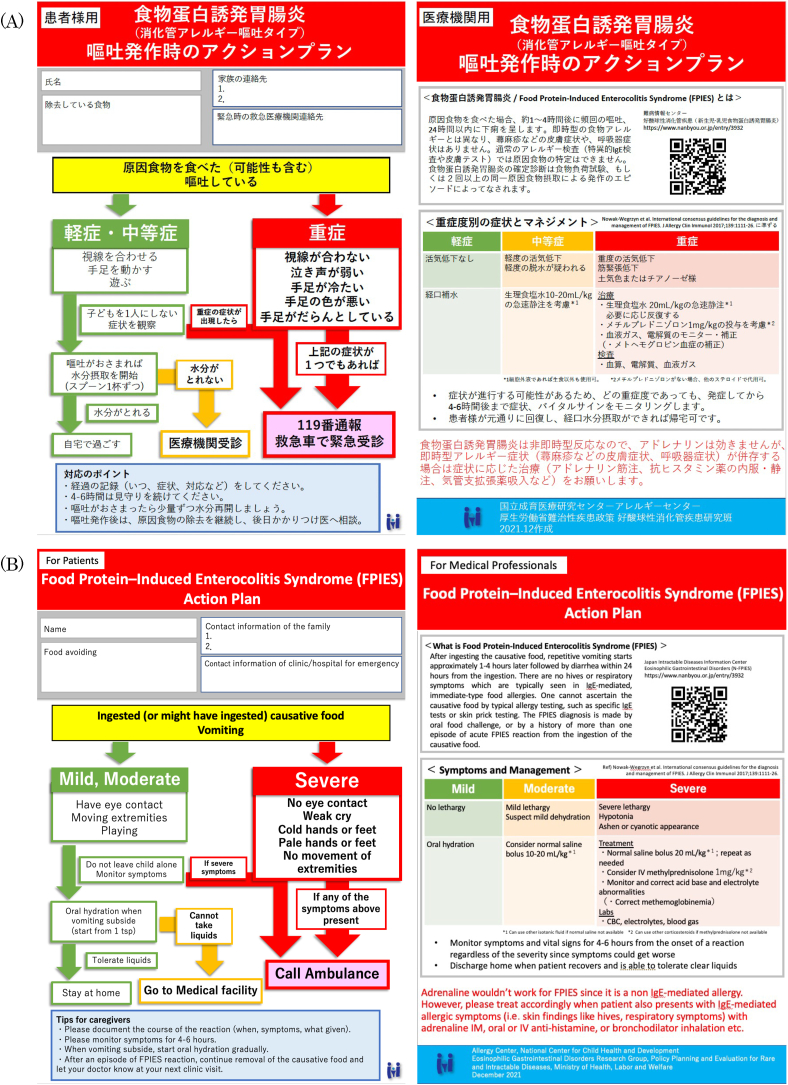
FPIES action plan (final version after the survey and consensus obtained). (A) Original in Japanese, (B) English translation.

Changes to the FPIES action plan were made based on the feedback from the participants of the survey (Fig. 1).

### Validation phase

To finalize the FPIES action plan and obtain consensus, the Delphi method was utilized.^11^, ^12^, ^13^ We created an online questionnaire evaluating content and wording, design, layout, and size, and overall quality of the action plan of both for patients and medical professionals sides (Supplemental Table 1). We used 5-point Likert scales, and “strongly agree” was grouped with “agree” and “strongly disagree” was grouped with “disagree.” The consensus on each question was defined as an agreement over 65%.^12^^,^^13^ The online questionnaire was distributed through email to participants including 20 caregivers and 28 physicians from several subspecialties and hospitals in different regions of Japan, though most were pediatricians (Supplemental Table 2). Caregivers were recruited through the same methodology from the development phase. Caregivers evaluated the “For Patients” side of the action plan and physicians evaluated both the “For Patients” and “For Medical Professionals” sides.

### Data analysis

Categorical data were reported as raw counts with percentages. For continuous data, mean ± standard deviation or median with interquartile range was used as appropriate.

## Results

### Development phase

A total of 30 physicians and 54 caregivers completed the questionnaire, and their background characteristics are shown in Table 1 and Supplemental Table 3, respectively. The median age of the physicians was 35 years old, the postgraduate year median was 10 years, and 60.0% of the physicians were male. Among all the physicians, 70.0% of them have seen patients with FPIES, and 43.3% of them had managed patients with acute FPIES reactions. The median age of the caregivers was 34 years old and most of them were mothers. In that group, 75.9% of caregivers had a college or higher level of education. The mean of Communicative and Critical Health Literacy scale was 3.5. Background characteristics of the patients is shown in Supplemental Table 4. The median patient age was 12 months old with the FPIES onset at 7 months old. Causal foods were egg yolk, egg white, soy, milk, and wheat in the order of majority. Thirteen percent (13.0%) of the patients had comorbid IgE-mediated food allergy.

**Table 1:** Background characteristics of the participating physicians for the initial survey

All the participating physicians (n = 30)	
Age, median (IQR)^a^	35 (30, 40)
PGY, median (IQR)^b^	10 (6, 15)
Male, n (%)	18 (60.0)
Subspecialty^c^	
Pediatrics, n (%)	29 (96.7)
Allergy, n (%)	7 (23.3)
Emergency medicine, n (%)	5 (16.7)
Gastroenterology, n (%)	1 (3.3)
ICU, n (%)	1 (3.3)
Number of FPIES patients they have seen	
0, n (%)	9 (30.0)
1–2, n (%)	10 (33.3)
3–5, n (%)	2 (6.7)
≥6, n (%)	9 (30.0)
**Physicians who have seen FPIES (n = 21)**	
Have you explained to patients what to do when they have acute FPIES reaction?	
Never, n (%)	7 (33.3)
Sometimes, n (%)	7 (33.3)
Always, n (%)	7 (33.3)
Have you managed patients with acute FPIES reaction?	
Yes, n (%)	13 (61.9)
No, n (%)	8 (38.1)
Management provided for acute FPIES reaction (n = 13)^c^	
Fluids, n (%)	12 (92.3)
Antiemetics, n (%)	3 (23.1)
Corticosteroids, n (%)	6 (46.2)
Admit patients, n (%)	9 (69.2)

PGY: postgraduate year.

a Missing value = 2.

b Missing value = 1.

c Multiple answers allowed

Supplemental Table 5 describes the ratings on the preliminary FPIES action by the physicians and the caregivers. They rated the action plan as easy to read and understand, but less easy to keep for future reference. The mean comprehensibility subtotal score among the physicians and the caregivers were 20.0 and 19.0 (score range 5–25), respectively. The mean design quality subtotal score among the physicians and the caregivers were 23.8 and 22.7 (score range 6–30), respectively. Most of the participants found the action plan to be useful and more than half of the participants rated the likelihood of using the action plan in the future high or very high.

Feedback in the open comments section was discussed among the authors, pediatric allergists, pediatric gastroenterologists, and pediatric emergency physician, and used to make corrections to the action plan accordingly as summarized in Supplemental Table 6. In particular, there were many comments regarding difficulty to distinguish between moderate and severe symptoms among caregivers. The action plan was modified to describe critical signs and symptoms of poor circulation and hypovolemic shock in severe symptoms in lay terms with the advice of the pediatric emergency physician, meanwhile combining mild and moderate signs and symptoms which describe good circulation. Importantly, after the discussion among the authors, the number of emesis was eliminated from the action plan as the management of acute FPIES reaction should be focused on hypovolemia and stabilizing patients and for caregivers to make the severity assessment easier. Another major change was to create the “Tips for caregivers” section, which explained what to do in general when patients have acute FPIES reaction or accidently ingested causal food but no reaction. In the international consensus guidelines of FPIES, patients with history of severe FPIES reaction are recommended to go to the emergency department regardless of symptoms.^2^ However, there was feedback from caregivers that this flow is difficult to understand. Since access to emergency medical care is relatively expedient in Japan, the authors eliminated the flow of going to emergency department for mild symptoms from the action plan. After all corrections were reviewed, the revised FPIES action plan was created (Fig. 1).

### Validation phase

One caregiver declined to participate in the Delphi study, so a total of 28 physicians and 19 caregivers participated in the study and answered all the questions (response rate 97.9%). Most of participants agreed to each statement on the revised FPIES action plan (Supplemental Table 6). After the first round, consensus was reached for all the statements per the Delphi method, and the FPIES action plan was finalized.

## Discussion

This single–sheet double-sided Japanese FPIES action plan, one side for caregivers and another side for medical professionals, was made with significant input from both caregivers and physicians. The Australasian Society of Clinical Immunology and Allergy (ASCIA) created a FPIES action plan which describes “mild to moderate symptoms” and “severe symptoms” and advises the reader that for mild to moderate symptoms, one should notify parent/guardians and observe for progression, and for severe symptoms to call an ambulance and the family/emergency contact.^14^ This action plan did not describe management steps for healthcare providers at medical facilities. Other action plan development is reported in the medical literature, but a copy of this finalized plan was not publicly available.^15^ To our knowledge, our FPIES action plan may be the first to describe management for both caregivers and medical professionals.

Our preliminary FPIES action plan was based on the international consensus guidelines of FPIES, but several modifications were required based on the results of the initial survey in this study. The final FPIES action plan in this study was well-received by the physicians and caregivers, but future modifications may still be needed as FPIES becomes better understood and more widely diagnosed.

There are several limitations to this study. Most of the participating caregivers were recruited at a single institution, so there is limited geographical variety in the participants. Over 70% of the caregivers had a college or higher level of education, which would limit generalizability to populations with lower socioeconomic status. The CIRF was developed in the United States and validated in Australia in English, but it was administered in Japanese in this study. Relative CIRF scores may vary across different cultures and languages. We did not repeat the CIRF or other questionnaire which were in the initial survey, so a comparison could not be performed between the pre- and post-modification scores. There was only one emergency medicine physician in the Delphi panel in this study. However, in Japan general pediatricians usually work at emergency rooms, and there are few pediatric emergency medicine physicians except for tertiary hospitals. Since the administration of ondansetron was excluded from this action plan, as this medication is not approved for FPIES in Japan, the usage of this action plan would be limited in other countries where ondansetron is prescribed for the management. We hope to develop an action plan that would be practical globally as a next step.

In summary, this Japanese FPIES action plan was created by physicians from multiple subspecialties and caregivers of patients with FPIES. Future goals include broadening this action plan usage across Japan nationwide so that patients with FPIES may receive appropriate treatment.

## Abbreviations

CIRF, Consumer Information Rating Form; FPIES, Food protein-induced enterocolitis syndrome; NCCHD, National Center for Child Health and Development.

## Acknowledgements

We would like to thank our research assistants, Kaori Moriwaki, Kumiko Watabe, and Kazuko Hayase and all the caregivers and the physicians who participated in the study.

## Funding

Health, Labor, and Welfare Sciences Research Grants for Research on Policy Planning and Evaluation for Rare and Intractable Diseases, from the 10.13039/100009647Ministry of Health, Labor and Welfare, Japan, Grant/Award Numbers: 20FC1016 (to I. Nomura), National Center for Child Heath and Development (grant number 2022E-1) and 10.13039/501100001691JSPS KAKENHI Grant Number JP 70839597, Japan.

## Availability of data and materials

The data of this study are not publicly available but are available from the corresponding authors upon reasonable request.

## Author contributions

KYH proposed the first research concept. YEK, MS, KYH, and IN developed the study design. YEK and MS prepared study results and contributed equally. YEK drafted the original manuscript. All co-authors contributed to modifying the FPIES action plan and the manuscript.

## Authors’ consent for publication

All authors read and approved the final manuscript for the publication.

## Ethics approval

This study was approved by the Institutional Review Board of the National Center for Child Health and Development (2021-050 and 2021-292).

## Declaration of competing interest

No competing interests related to the contents of this study.
