# Supplementary material for: Development of an action plan for acute food protein–induced enterocolitis syndrome in Japan
Source: World Allergy Organ J. 2023 May 9;16(5):100772. doi: 10.1016/j.waojou.2023.100772 (PMC10282562; doi:10.1016/j.waojou.2023.100772)
Supplement: Multimedia component 1 [file mmc1.pdf]

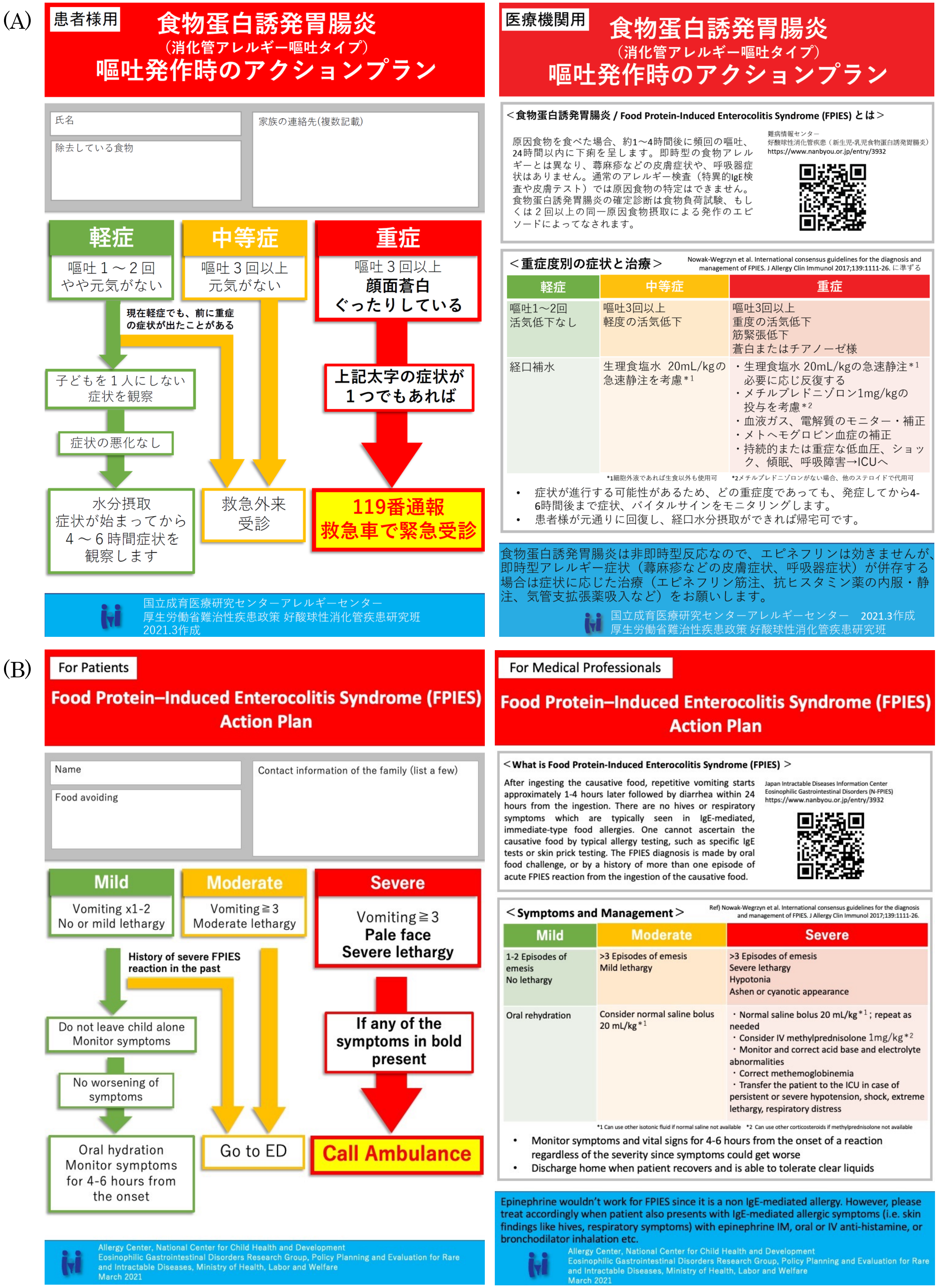

Supplemental Fig 1. FPIES action plan (preliminary version before the survey)
(A) Original in Japanese, (B) English translation
