# Supplementary material for: Development of an action plan for acute food protein–induced enterocolitis syndrome in Japan
Source: World Allergy Organ J. 2023 May 9;16(5):100772. doi: 10.1016/j.waojou.2023.100772 (PMC10282562; doi:10.1016/j.waojou.2023.100772)
Supplement: Multimedia component 2 [file mmc2.docx]

Supplemental Table 1. Delphi study questions and responses

| **Questions** | **Responses, n (%)** | | |
| --- | --- | --- | --- |
|  | **Yes^a^** | **Neither** | **No^b^** |
| **“For patients” side** | **n = 47** | | |
| Content and wording  Q1. Is it easy to understand the difference between the mild and moderate symptoms and severe symptoms?  Q2. Is it easy to understand what to do when symptoms occur? | 45 (95.7)  43 (91.5) | 1 (2.1)  3 (6.4) | 1 (2.1)  1 (2.1) |
| Design, layout, and size  Q3. Is the design and layout appropriate?  Q4. In addition to distributing as an electronic file (such as QR code, etc.), printing in a business card size format when folded is being considered. Is this size easy to carry around? | 44 (93.6)  44 (93.6) | 1 (2.1)  0 (0) | 2 (4.3)  3 (6.4) |
| Overall  Q5. Do you agree to the “For patients” side of the FPIES action plan? | 45 (95.7) | 2 (4.3) | 0 (0) |
| **“For medical professionals” side** | **n = 28** | | |
| Content and wording  Q1. Is the expression of the symptoms of each severity appropriate?  Q2. Is the management of each severity appropriate?  Q3. Is the expression in the red, “Epinephrine wouldn’t work for FPIES since it is a non IgE-mediated allergy…” appropriate? | 25 (89.3)  25 (89.3)  25 (89.3) | 1 (3.6)  1 (3.6)  2 (7.1) | 2 (7.1)  2 (7.1)  1 (3.6) |
| Design and layout  Q4. Is the design and layout appropriate? | 26 (92.9) | 2 (7.1) | 0 (0) |
| Overall  Q5. Do you agree to the “For medical professionals” side of the FPIES action plan? | 26 (92.9) | 2 (7.1) | 0 (0) |

^a^Yes includes “strongly agree” and “agree.” ^b^No includes “strongly disagree” and “disagree.”

Supplemental Table 2. Characteristics of the participants for the Delphi study (n = 47)

| Physicians’ subspecialty^a^ (n = 28) | |
| --- | --- |
| Pediatrics, n (%) | 27 (96.4) |
| Allergy, n (%) | 10 (35.7) |
| Emergency medicine, n (%) | 1 (3.6) |
| Gastroenterology, n (%) | 3 (10.7) |
| **Caregivers (n = 19)** | |
| Mother, n (%) | 17 (89.5) |
| Father, n (%) | 2 (10.5) |

^a^multiple answers allowed

Supplemental Table 3. Background characteristics of the participating caregivers for the initial survey (n = 54)

|  |  |
| --- | --- |
| Age, median (IQR)^a^ | 34 (30, 38) |
| Answerer, n (%) |  |
| Mother | 50 (92.6) |
| Father | 4 (7.4) |
| Education | |
| High school, n (%) | 2 (3.7) |
| Vocational school, n (%) | 6 (11.1) |
| Two-year college, n (%) | 4 (7.4) |
| College, n (%) | 35 (64.8) |
| Master, PhD, n (%) | 6 (11.1) |
| No answer | 1 (1.9) |
| Communicative and Critical Health Literacy scale, mean (SD) | 3.5 (0.67) |

^a^missing value = 1

Supplemental Table 4. Background characteristics of the patients for the initial survey (n = 54)

|  | | |
| --- | --- | --- |
| Age (months old), median (IQR) | | 12 (11, 18) |
| Age of FPIES onset (months old), median (IQR)^a^ | | 7 (6, 8) |
| Male, n (%) | | 22 (40.7) |
| Number of the acute FPIES episodes | | |
| 1, n (%) | | 1 (1.9) |
| 2, n (%) | | 22 (40.7) |
| <=3, n (%) | | 31 (57.4) |
| Severity of FPIES | | |
| Mild, n (%) | | 18 (33.3) |
| Moderate, n (%) | | 17 (31.5) |
| Severe, n (%) | | 19 (35.2) |
| ED visit due to FPIES, n (%) | | 31 (57.4) |
| Hospitalization due to FPIES, n (%) | | 11 (20.4) |
| Causal food^b^ | | |
| Egg yolk, n (%) | | 43 (79.6) |
| Egg white, n (%) | | 11 (20.4) |
| Soy, n (%) | | 8 (14.8) |
| Milk, n (%) | | 4 (7.4) |
| Wheat, n (%) | | 3 (5.6) |
| Rice, n (%) | | 0 (0) |
| Other allergic comorbidities, n (%)^b^ | | 9 (16.7) |
| IgE-mediated food allergy | | 7 (13.0) |
| Atopic dermatitis | | 5 (9.3) |
| Allergic rhinitis, conjunctivitis | | 2 (3.7) |
| Asthma | | 0 (0) |
| Allergic disease in the father^b^ | | 22 (40.7) |
| IgE-mediated food allergy | | 5 (9.3) |
| Atopic dermatitis | | 5 (9.3) |
| Allergic rhinitis, conjunctivitis | | 21 (38.9) |
| Asthma | | 2 (3.7) |
| FPIES | | 0 (0) |
| Eosinophilic gastrointestinal disorders | | 0 (0) |
| Allergic disease in the mother^b^ | | 27 (50.0) |
| IgE-mediated food allergy | | 5 (9.3) |
| Atopic dermatitis | | 3 (5.6) |
| Allergic rhinitis, conjunctivitis | | 24 (44.4) |
| Asthma | | 5 (9.3) |
| FPIES | | 0 (0) |
| Eosinophilic gastrointestinal disorders | | 0 (0) |
|  |  | |

^a^missing value = 1, ^b^multiple answers allowed

Supplemental Table 5. Ratings on the preliminary FPIES action plan by the physicians and the caregivers

| CIRF items | Physicians (n = 30) | Caregivers (n = 54) |
| --- | --- | --- |
| Comprehensibility, mean ± SD  (1 = very hard, 5 = very easy) |  |  |
| Read | 4.2 ± 0.77 | 4.1 ± 0.75 |
| Understand | 4.2 ± 0.77 | 4.0 ± 0.89 |
| Remember | 4.0 ± 0.81 | 3.8 ± 0.74 |
| Find information | 3.9 ± 0.91 | 3.9 ± 0.73 |
| Keep | 3.7 ± 1.0 | 3.3 ± 1.0 |
| Subtotal score (range 5-25) | 20.0 ± 3.4 | 19.0 ± 3.1 |
| Design quality, mean ± SD  (1 = very low, 5 = very high) |  |  |
| Organization | 4.1 ± 0.76 | 3.8 ± 0.74 |
| Attractiveness | 4.0 ± 0.83 | 3.7 ± 0.83 |
| Print size | 4.1 ± 0.83 | 3.8 ± 0.80 |
| Tone | 4.0 ± 0.79 | 3.7 ± 0.81 |
| Helpfulness | 3.9 ± 0.86 | 3.9 ± 0.75 |
| Spacing | 3.8 ± 0.90 | 3.7 ± 0.79 |
| Subtotal score (range 6-30) | 23.8 ± 4.1 | 22.7 ± 3.9 |
| **Other items** |  |  |
| Usefulness, n (%) |  |  |
| Very useful | 13 (43.3) | 21 (38.9) |
| Useful | 11 (36.7) | 26 (48.1) |
| Neither useful nor useless | 6 (20) | 6 (11.1) |
| Useless | 0 (0) | 1 (1.9) |
| Very useless | 0 (0) | 0 (0) |
| Improvements in:  (1 = no changes, 5 = very much improved) |  |  |
| Knowledge, mean ± SD | 3.5 ± 0.97 | 3.6 ± 0.95 |
| Understanding, mean ± SD | 3.5 ± 0.90 | 3.5 ± 0.93 |
| Likelihood of using in the future, n (%) |  |  |
| Very high | 10 (33.3) | 9 (16.7) |
| High | 13 (43.3) | 24 (44.4) |
| Not sure | 6 (20) | 18 (33.3) |
| Low | 1 (3.3) | 1 (1.9) |
| Very low | 0 (0) | 2 (3.7) |

CIRF: Consumer Information Rating Form Supplemental Table 6. Summary of feedback on the preliminary FPIES action plan by the physicians and the caregivers and corresponding corrections

| **Feedback** | **Corrections** |
| --- | --- |
| **“For patients” side** | |
| - Difficult to tell the difference between mild, moderate and severe lethargy, especially between moderate and severe since both are vomiting more than twice | - Deleted the word “lethargy” and explained more specifically about symptoms of different severity - Deleted the number of vomiting to distinguish severity of symptoms |
| - What to do when the child ingests causal food but no symptoms? - What to do when there is worsening of symptoms in mild reaction? | - Added time course to the flow |
| - What do you mean by “worsening of symptoms” in the flow of mild reaction? | - Added flowchart “If severe symptoms” |
| - Hard to understand the flow of “History of severe FPIES reaction in the past” | - Deleted the flow |
| - What to do when monitoring at home? - What to do after monitoring for 4-6 hours at home? - What to do until going to medical facility? | - Made a section “Tips for caregivers” |
| - How much fluid to give at home? | - Added a comment to start from 1 tsp |
| - Request a section for contact information of medical facility for emergency | - Added a box to write down contact information of clinic/hospital for emergency |
| **“For medical professionals” side** | |
| - The appearance and circulation should be prioritized over the frequency of emesis | - Deleted the number of vomiting to distinguish severity of symptoms |
| - The section on the management of severe cases does not look organized | - Separated the treatment and work-up in the management section - Deleted the criteria for ICU admission |
| - What labs to order in severe cases? | - Added what labs to order |
| - What is the treatment for methemoglobinemia? | - Described the treatment on the website that QR code on the action plan directs to - Put parenthesis since it’s not common |
| - Treatment with normal saline bolus 20 ml/kg is different from PALS 10-20 ml/kg | - Changed to normal saline 10-20 ml/kg in the treatment for moderate symptoms |
| - The word “Epinephrine” is not appropriate | - Changed to “Adrenaline” |
| - The sentence at the bottom about concomitant IgE-mediated allergy is hard to read | - Changed format and coloring |
| **As a whole** | |
| - A4 size is not portable | - Plan to print in business card size when folded and also to distribute electronically |
